# Waste-to-wealth application of wastewater treatment algae-derived hydrochar for Pb(II) adsorption

**DOI:** 10.1016/j.mex.2021.101263

**Published:** 2021-02-07

**Authors:** Jiuling Yu, Tianbai Tang, Feng Cheng, Di Huang, Julia L. Martin, Catherine E. Brewer, Ronald L. Grimm, Meng Zhou, Hongmei Luo

**Affiliations:** a Department of Chemical and Materials Engineering, New Mexico State University, Las Cruces, NM 88003, USA; b Department of Chemistry and Biochemistry, Life Science and Bioengineering Center, Worcester Polytechnic Institute, 100 Institute Road, Worcester, Massachusetts 01609, USA

**Keywords:** Microalgae, hydrothermal liquefaction, char activation, heavy metal adsorption

## Abstract

Hydrochar, as an energy-lean solid waste, is generated from an advanced biofuel conversion technique hydrothermal liquefaction (HTL) and always leads to environmental pollution without appropriate disposal. In this study, HTL-derived hydrochar is recycled and prepared as adsorbent used for Pb(Ⅱ) removal from wastewater. As the original porous structure of hydrochar is masked by oily volatiles remained after HTL, two types of oil-removal pretreatment (Soxhlet extraction and CO_2_ activation) are explored. The result shows that CO_2_ activation significantly enhances the adsorption capacity of Pb(Ⅱ), and the maximum adsorption capacity is 12.88 mg g^−1^, as evaluated using Langmuir adsorption model. Further, apart from oily volatiles, most inorganic compounds derived from wastewater-grown algae is enriched in hydrochar, causing a smaller surface area of hydrochar. An ash-removal alkali treatment following CO_2_ activation is introduced to dramatically increase the adsorption capacity to 25.00 mg g^−1^ with an extremely low Pb(II) equilibrium concentration of 5.1×10^-4^ mg L^−1^, which is much lower than the maximum level of Pb concentration in drinking water (set by World Health Organization). This work introduces an approach to reuse HTL-hydrochar as an inexpensive adsorbent in Pb-contaminated water treatment, which not only provides another possible renewable adsorbent candidate applied in the field of lead adsorption, but also finds an alternative route to reduce solid waste effluent from HTL process.

## Graphical abstract

**Figure:**
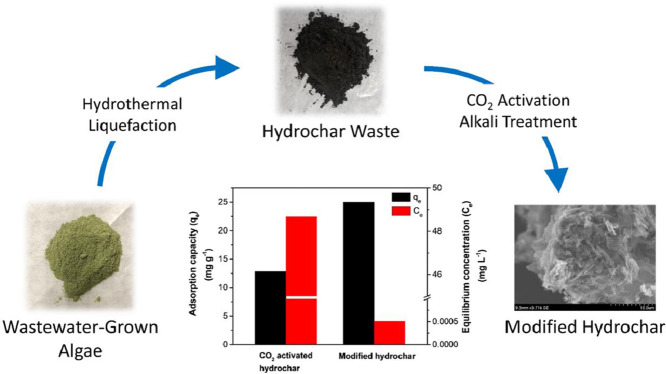

Specifications table

**Table:** 

Subject Area	Environmental Science
More specific subject area	Adsorption
Method name:	Pb(II) adsorption using the hydrochar produced from hydrothermal liquefaction of microalgae
Protocol name	Waste-to-wealth application of wastewater treatment algae-derived hydrochar for Pb(II) adsorption
Reagents/tools	A list of reagents: • Acetone (CH_3_COCH_3_, ≥99.5%) • Sodium hydroxide (NaOH, 99.99%) • Sodium bicarbonate (NaHCO_3_, ≥99.7%) • Sodium carbonate (Na_2_CO_3_, ≥99.0%) • Lead nitrate (Pb(NO_3_)_2_, ≥99.0%) • Nitric acid (HNO_3_, 70%) • Sulfuric acid (H_2_SO_4_, 95–98.0%) A list of equipment: • Series II 2400 elemental analyzer (Perkin Elmer) • Model 6725 semi-micro bomb calorimeter (Parr Instrument Company) • TGA-Q500 (TA Instruments) • Elan DRC-E inductively coupled plasma mass spectrometer (PerkinElmer) • Spectrum Two FT-IR spectrophotometer (PerkinElmer) • S-3400 N II scanning electron microscope (Hitachi) • PHI 5600 XPS system
Experimental design	Oil extraction and CO_2_ activation were performed on the hydrochar produced from HTL process. The effect of initial pH and concentration were investigated. Alkali treatment was used to increase the adsorption capacity.
Trial registration	*Not applicable*
Ethics	*Not applicable*
Value of the Protocol	• Byproduct HTL hydrochar is evaluated as Pb(II) adsorbent in the wastewater treatment. • CO_2_ activation removes organic compounds in the HTL process and increases adsorption capacity of Pb(II) to 12.88 mg g^−1^ based on Langmuir model. • One-step alkali treatment further enhances the adsorption capacity to 25.00 mg g^−1^ with very low equilibrium concentration of 5.1×10^-4^ mg L^−1^. • HTL hydrochar shows the feasibility as the inexpensive adsorbent for Pb(II) removal.

## Protocol background

While lead (Pb) has been commonly used throughout human history, its toxicity and non-biodegradability have received additional attention in recent years. Large quantities of Pb(II)-containing effluent, generated from smelting, painting, battery production, and ceramic/glass manufacturing [1], [2], [3], [4], are released into groundwater, threatening the neurological, cardiovascular, and renal systems of humans through binding with sulfhydryl groups on proteins [5,6]. The World Health Organization recommends that the concentration of lead in drinking-water should not exceed 10 μg L^−1^; and the U.S. Environmental Protection Agency requires that lead-ion concentration of effluents should be reduced to <50 μg L^−1^ before discharge [7].

To date, several traditional techniques have been reported for Pb(II) removal from water: coagulation-flocculation [8], membrane filtration [9], ion-exchange [10], and adsorption [11]. The low cost and simple operation requirements for adsorption make it an attractive strategy. The precursors used to produce commercial adsorbent (e.g. activated carbon) generally come from fossil fuels, such as coal and petroleum [12]. Nevertheless, with the excessive depletion of fossil fuels, seeking for the sustainable and green precursors is crucial. As the plentiful and easy-accessible resource, biomass has been widely used as origin to produce the inexpensive adsorbent. Recently, carbons produced from a broad range of biomass, such as watermelon peel [2], grape pomace [13], sugarcane bagasse [14], and pinewood [15], have been found to be highly effective adsorbents for Pb(II) removal in wastewater treatment. Currently, most biomass derived carbons are synthesized through hydrothermal carbonization (HTC) or slow pyrolysis (SP) method, in which the char is the main product. Unlike HTC or SP, hydrothermal liquefaction (HTL) is a promising route to generate bio-oil as primary product [16]. In the meanwhile, hydrochar can be obtained from HTL as byproduct with yield ranging from 20 to 40% [17], which unfortunately has negative effect on the economic benefit. Hence, it is worthwhile finding a profitable way to reutilize HTL-hydrochar other than directly disposing as waste. Recently, HTL-hydrochar was used as efficient adsorbent to remove organic pollutant (e.g. methylene blue dye) in the wastewater [18]. Besides organic contaminant, the adsorption performance of HTL-hydrochar on heavy metal pollutant, such as Pb(II), is worthy to investigate as well. Another interesting point is that most papers focus on high adsorption capacity of heavy metals, rather than discussing equilibrium concentration. However, high uptake at equilibrium might not reach the safe disposal rule on the concentration.

Herein, this work evaluates Pb(II) adsorption ability and equilibrium concentration using the hydrochar generated from HTL of wastewater-grown filamentous algae. However, low surface area, low porosity, heavy organic compound content, and ash/metal oxide content of the wastewater algae-derived hydrochar are challenges for use of the hydrochar as a high-efficiency adsorbent [19]. Therefore, two pretreatments (oil extraction and CO_2_ activation) were performed so as to investigate the effects on the Pb(II) adsorption capacity. Furthermore, considering the high content of ash accumulated in the hydrochar derived from wastewater-grown algae, a simple step of alkali treatment was employed to study the effect of ash removal on Pb(II) adsorption. Thereby, in this study, Pb(II) adsorption capacity of wastewater algae-derived hydrochar was evaluated as treated with oil extraction, CO_2_ activation, and alkali treatment to optimize the Pb(II) adsorption condition. The adsorption capacity is not only simulated by Langmuir and Freundlich models, but also evaluated on the Pb(II) concentration at equilibrium.

## Materials and methods

### Materials

Acetone (CH_3_COCH_3_, ≥99.5%), sodium hydroxide (NaOH, 99.99%), sodium bicarbonate (NaHCO_3_, ≥99.7%), sodium carbonate (Na_2_CO_3_, ≥99.0%), lead nitrate (Pb(NO_3_)_2_, ≥99.0%), nitric acid (HNO_3_, 70%), and sulfuric acid (H_2_SO_4_, 95–98.0%) were purchased from Sigma-Aldrich. All the chemicals were used as received.

### Hydrochar pretreatment and alkali treatment

Untreated hydrochar (U_char) was produced from wastewater-grown filamentous microalgae via HTL process. The operation condition was 10 wt.% algal solids loading at 350 °C for 30 min. The detailed experiments are shown in the previous work [20].

Two types of hydrochar pretreatment, oil extraction and CO_2_ activation, were performed prior to adsorption experiments. For oil extraction, heavier organic compounds were removed from hydrochar using 175 mL acetone at 56 °C in a Soxhlet extractor until acetone color turns transparent in the extractor. As for CO_2_ activation, hydrochar (0.5 g) was heated to 800 °C at 5 °C min^−1^ ramping and then kept for 45 min under 30 cm^3^ min^−1^ CO_2_ flow. The combination of the two pretreatments was tested for a total of four types of hydrochars: untreated (U_char), oil extracted (EN_char), CO_2_ activated (UA_char), and oil extracted and CO_2_ activated (EA_char). To further remove ash, CO_2_-activated char was mixed and stirred in a 5 M NaOH solution at 60 °C, followed by filtering with DI water to obtain neutral pH and drying in the vacuum oven overnight. “NaOH-washed” CO_2_-activated char was denoted as UAW_char.

### Batch adsorption of Pb(II) onto hydrochar

To evaluate the capacity of Pb(II) adsorption, a stock Pb(II) solution (1000 mg L^−1^) was prepared by dissolving Pb(NO_3_)_2_ in DI water, from which the test Pb solutions (10, 20, 30, and 100 mg L^−1^) were prepared by dilution with DI water. HNO_3_ or NaOH were used to adjust the pH of the Pb solutions. Batch adsorption experiments were performed by mixing each hydrochar (100 mg) with 25 mL of the prepared Pb solutions with continuous magnetic stirring at 25 °C for 5 h. All adsorption experiments were carried out in duplicate. The adsorption capacity was estimated using [2]:

(1) $$q_{e}=\frac{\left(C_{0}-C_{e}\right)\times V}{W}$$

The percentage of removed Pb(II) was estimated by [21]:

(2) $$\text{Percentage}\;\text{removal}\;\left(\%\right)=\frac{\left(C_{0}-C_{e}\right)}{C_{0}}\times \;100$$

where *q*_e_ (mg Pb g^−1^ adsorbent) is the adsorption capacity at equilibrium, *C*_0_ (mg L^−1^) and *C*_e_ (mg L^−1^) are the initial concentration and equilibrium concentrations, respectively, *V* (L) is the volume of Pb(II) solution, and *W* (g) is mass of adsorbent added.

The Langmuir [22] and the Freundlich [23] adsorption models were compared with the adsorption data, using the linearized forms [24], here:

(3) $$\text{Langmuir}\;\frac{C_{e}}{q_{e}}=\frac{1}{K_{L}q_{m}}+\frac{C_{e}}{q_{m}}\;$$

(4) $$\text{Freundlich}\;\text{In}\;q_{e}=\text{In}\;K_{F}+\frac{1}{n}\text{In}\;C_{e}$$

where *C*_e_ (mg L^−1^) is the equilibrium Pb concentration in solution, *q*_e_ (mg g^−1^) is Pb(II) adsorbed at equilibrium, *q*_m_ (mg g^−1^) is the maximum adsorption capacity, *K*_L_ (L mg^−1^) is the Langmuir constant, *K*_F_ (mg g^−1^) is the Freundlich constant related to adsorption capacity, and *n* is the constant related to energy.

In particular, UA_char and UAW_char were named as UAP_char and UAWP_char, respectively, after Pb(II) adsorption.

### Hydrochar characterization

The elemental CHNS content was analyzed by a Series II 2400 elemental analyzer from Perkin Elmer. Higher heating values (HHV) of the feedstock and hydrochar were measured using a Model 6725 semi-micro bomb calorimeter (Parr Instrument Company). Thermogravimetric analysis (TGA) was used to determine the ash content and performed on the TGA-Q500 from TA Instruments. All samples were heated up to 800 °C from room temperature (25 °C) with the ramping rate of 10 °C min^−1^ under air atmosphere (60 mL min^−1^). Oxygen content was calculated by difference. Solution Pb concentration was measured by a PerkinElmer Elan DRC-E inductively coupled plasma mass spectrometer (ICP-MS). All above measurements were carried out in duplicate. To study the chemical functional groups on the hydrochar surfaces, Fourier-transform infrared spectroscopy (FT-IR) were performed on a Spectrum Two FT-IR spectrophotometer (PerkinElmer). Oxygen-containing acidic surface functional groups were estimated by Boehm titration [25]; details can be found in the Supplementary material. To characterize the surface morphologies of samples, scanning electron microscopy (SEM) were examined on a S-3400 N II scanning electron microscope. Surface elemental composition was analyzed by X-Ray photoelectron spectroscopy (XPS) on a PHI 5600 XPS system as described previously [26]; detailed information is described in the Supplementary material.

## Results and discussion

### Characterization of algae and hydrochar

The composition of the algal biomass and untreated hydrochar (U_char) [27] are shown in Table 1. Ash content in U_char is substantially higher but carbon content lower than in the algal biomass, which is ascribed to the accumulation of majority of water- and oil-insoluble ash into solid phase and extraction of carbon-rich organics into bio-crude oil during HTL process [27,28]. Ash-rich U_char, thereby, has lower HHV than that of algal biomass. On a dry ash-free basis, the carbon content of U_char (46.4 wt.%) is similar to that of algal biomass (46.6 wt.%). The O/C ratio of U_char (0.85) is higher than that of feedstock (0.75), implying a certain amount of oxygen-containing functional groups may exist on the U_char surface [29]. One of our previous studies on inorganic element analysis [30] of algal biomass (Table S1) shows that ash component is enriched into U_char during HTL of wastewater algae.

**Table 1:** Composition of algal biomass feedstock and untreated hydrochar (U_char). Values are the average ± standard deviation of duplicate measurements.

	Algal biomass	U_char
Proximate analysis^a^		
Ash content (wt.%)	13.5 ± 0.4	41.8 ± 0.5^c^
HHV (MJ kg^−1^)	26.2 ± 0.3	20.3 ± 0.5
Elemental analysis (wt.%)^a^		
Carbon	40.3 ± 1.5	27.0 ± 0.3^c^
Hydrogen	6.1 ± 0.2	1.7 ± 0.3^c^
Nitrogen	7.1 ± 0.5	2.7 ± 0.1^c^
Sulfur	2.6 ± 0.3	3.9 ± 1.5^c^
Oxygen^b^	30.4 ± 1.7	22.9 ± 1.6

a dry basis

b by difference

c data is obtained from [27].

**Fig. 1:**
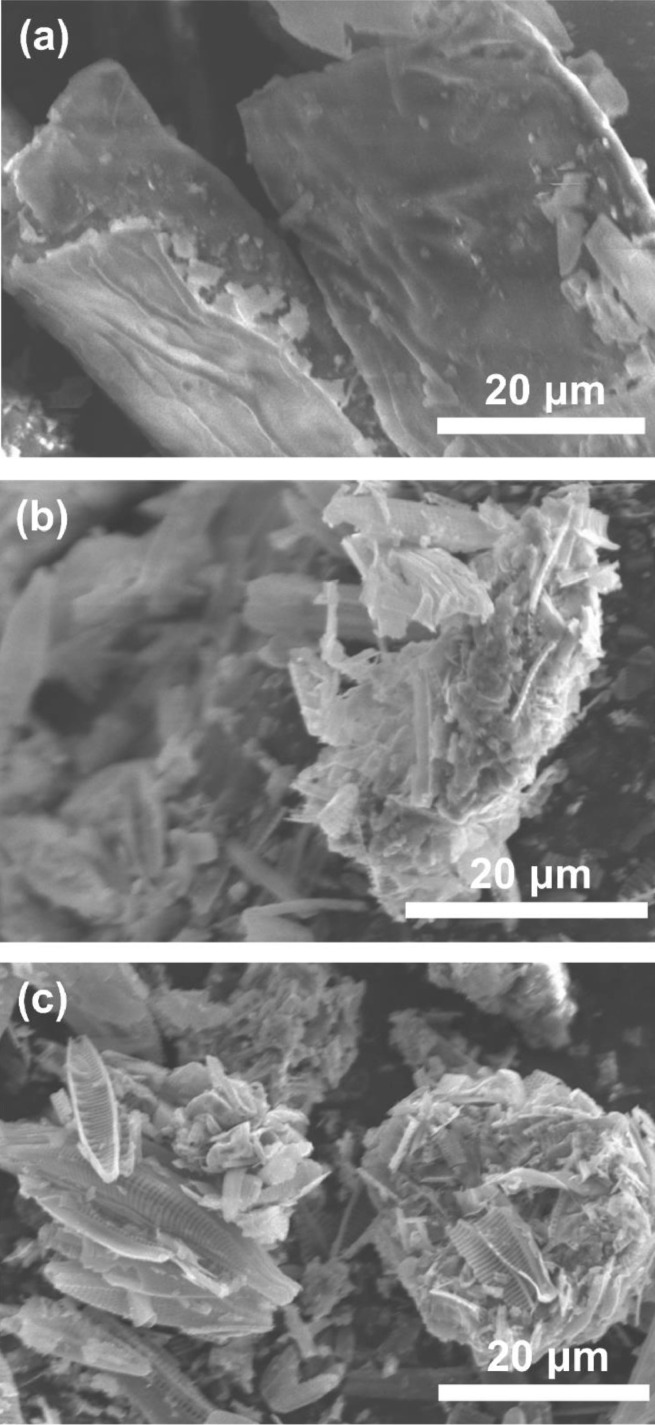
SEM images of (a) algal biomass; (b) U_char; and (c) UA_char.

Surface structural differences between algae, U_char and CO_2_-activated hydrochar (UA_char) are shown in Fig. 1. The filamentous algal biomass in Fig. 1(a) shows flat and smooth surface in a rectangular-type structure. In contrast, U_char and UA_char show irregular aggregation consisting of numerous small cracks (Fig. 1(b,c)), due to the volatiles released during HTL [18]. UA_char has more lance-shaped structures, while there is less porous structure observed on the U_char surface. The high temperature during CO_2_ activation causes additional oxidation/volatilization of organics from algal biomass, resulting in formation of more porous structures and exposure of more active sites. These newly-formed porous structures provide more paths for Pb(II) ion diffusion; the larger surface area provides more binding sites for Pb(II) ion adsorption [13].

**Fig. 2:**
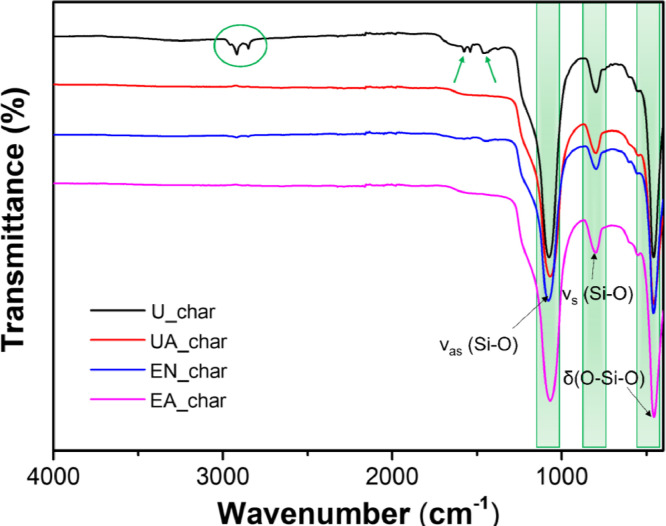
FT-IR spectra of U_char, UA_char, EN_char, and EA_char (FT-IR spectra of U_char and UA_char are obtained from our previous paper [27]).

FT-IR spectra of U_char [27], UA_char [27], oil-extracted hydrochar (EN_char), and oil-extracted and CO_2_-activated hydrochar (EA_char) are shown in Fig. 2. Compared to the other types of hydrochar, U_char exhibits several additional bands at 2916, 2850, 1582, 1538, and 1453 cm^−1^. Specifically, methyl C—H and methylene C—H stretching vibrations are identified at 2916 and 2850 cm^−1^ [27]. The small peaks at 1582, 1538, and 1453 cm^−1^ are contributed to the aromatic *C* = *O* stretching, aromatic *C* = *C* stretching and CH_2_ bending, respectively [31]. No methyl, methylene or aromatic peaks are found in UA_char, EN_char and EA_char, indicating that both oil extraction and CO_2_ activation may remove methyl, methylene, or aromatic groups from the char surface. The EA_char exhibits flat spectra compared with U_char, implying few functional groups (in particular oxygen-containing groups) remain on the char surface, attributed to the temperature of 350 °C during HTL conditions and post-HTL treatments. For instance, carboxylic and lactone groups decompose easily between 200 and 800 °C [32]. Interestingly, three strong peaks in the range of 450–1100 cm^−1^ are observed in all hydrochar samples, suggesting the presence of silica in the hydrochar [33], which is in agreement with the amount of ash (Table 1). Specifically, the sharp peaks at 1075, 797, and 456 cm^−1^ [27] are assigned to asymmetric Si-O stretching vibration, symmetric Si-O stretching motion [34,35], and O-Si-O bending vibration [36,37], respectively. This result implies two pretreatments of oil extraction and CO_2_ activation could effectively remove the insoluble organic function groups during HTL process from the hydrochar sample.

Table 2 shows that the concentrations of the three groups of organic acids (carboxyl, lactone, and phenolic hydroxyl) are very low in all hydrochar samples. The total acidity is in the range of 0.06 to 0.13 mmol g^−1^, which is significantly smaller than that for char obtained under hydrothermal carbonization conditions [38,39]. This is consistent with the observations from FT-IR in Fig. 2. Total acidities of UA_char and EA_char decrease by 27% and 54% in comparison with U_char and EN_char, respectively, which implies that further carbonization occurs during CO_2_ activation [40].

**Table 2:** Contents of acidic oxygen-containing functional groups for U_char, UA_char, EN_char, and EA_char.

Sample	Acidic surface functional group (mmol *g* ^−^ ^1^)			
	Carboxyl^a^ (–COOH)	Lactone^b^ (–*C* = *O*)	Phenolic hydroxyl^c^ (–OH)	Total acidity
U_char	0.01	0.06	0.04	0.11
UA_char	0.01	0.02	0.05	0.08
EN_char	0.04	0.07	0.02	0.13
EA_char	0.00	0.01	0.05	0.06

a estimated by the consumption of NaHCO_3_,

b estimated by the difference value between the consumption of Na_2_CO_3_ and NaHCO_3_,

c estimated by the difference value between the consumption of NaOH and Na_2_CO_3_.

### Hydrochar Pb(II) adsorption capacity

**Fig. 3:**
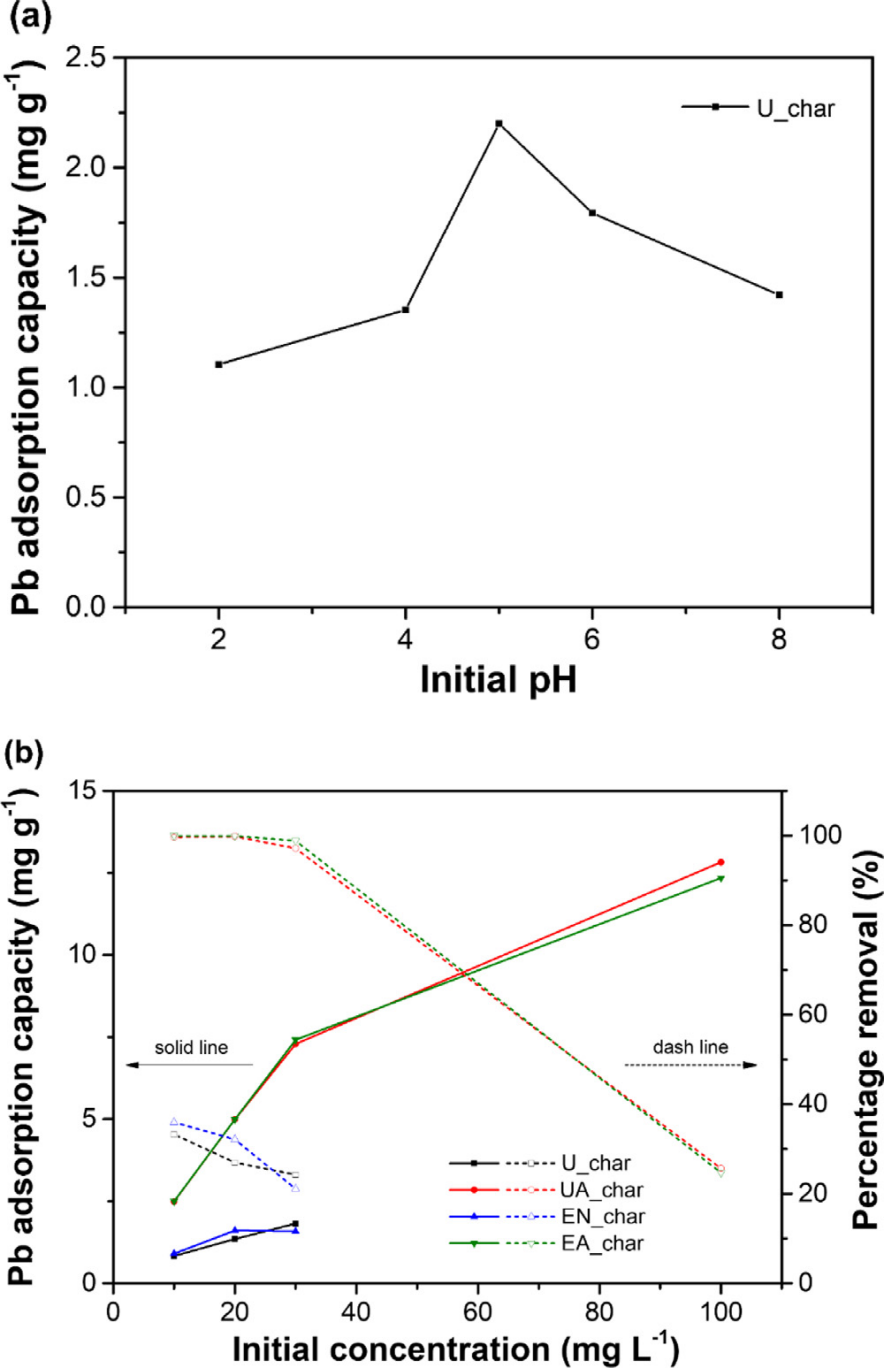
Effects of (a) initial solution pH on Pb(II) adsorption onto U_char; and (b) initial Pb(II) concentration on Pb adsorption capacity in terms of Pb(II) removal percentage for U_char, UA_char, EN_char, and EA_char. Adsorption conditions were 4 g L^−1^ adsorbent and room temperature.

In the metal adsorption, there is a strong correlation between solution pH and formation of metal ions and surface charges on the adsorbent [41], and the degree of ionization for the adsorbate [42]. The effect of initial pH on Pb(II) adsorption onto U_char is shown in Fig. 3(a). The adsorption capacity increases with pH from 2.0 to 5.0 and then decreases as pH > 5.0, with a maximum adsorption capacity of 2.2 mg g^−1^ at pH 5.0. At pH < 5.0, Pb(II) speciation favors the ionic state, resulting in a competition between Pb(II) and protons in the solution for sites on the hydrochar surface [43]. Increasing pH value deprotonates occupied active sites, which is beneficial to Pb^2+^ adsorption, leading to an improvement in adsorption capacity. Pb(II), however, precipitates into Pb(OH)_2_ as pH exceeds 6.0 [44,45]. Thus, all adsorption experiments are carried out at pH 5.0.

Another essential factor in the adsorption process is the initial concentration metal ions, which affects equilibrium with the adsorbent [46] and the adsorption rate [47]. Fig. 3(b) shows a clear correlation between adsorption capacities and initial concentrations of Pb(II) for U_char, UA_char, EN_char, and EA_char. Adsorption capacities of all hydrochar samples increase with initial Pb concentrations. The increase in the adsorption capacity from 10 to 30 mg L^−1^ is owing to transferring of Pb(II) ions into the interior structure of hydrochar facilitated by greater concentration gradients of Pb(II) ions between the solution and the char. At low initial concentration, Pb(II) ions are mainly adsorbed onto the outer surface of hydrochar, resulting in weaker adsorption capacity [48]. The capacities of the U_char and EN_char are identical for initial Pb concentrations from 10 to 30 mg L^−1^. This indicates that oil extraction had no remarkable impact on Pb(II) adsorption. The adsorption capacity of UA_char is substantially 3.0, 3.7, and 4.0 times higher than U_char at 10, 20, and 30 mg L^−1^, respectively. Similarly, the adsorption capability of EA_char is 2.8, 3.1, and 4.7 times greater than that of EN_char at 10, 20, and 30 mg L^−1^, respectively. This indicates that more adsorption sites were formed on hydrochar surface during CO_2_ activation. It is worth noting that the percentages of Pb(II) removal for UA_char and EA_char are close to 100% at low initial Pb(II) concentrations. As the initial concentration increases to 100 mg L^−1^, the adsorption capacity of UA_char and EA_char reaches 12.83 and 12.34 mg g^−1^, respectively, whereas their removal ratios decrease to 25.7% and 24.7%, indicating that the adsorption sites on CO_2_-activated hydrochar are fully saturated.

Fig. S1 shows the linear isotherm plots for Langmuir and Freundlich models, respectively. The values of the Langmuir and Freundlich isotherm parameters with corresponding correlation coefficients (*R*^2^) are summarized in Table 3. *R*^2^ values for the Langmuir model are higher than those for the Freundlich model, indicating that the Langmuir model describes Pb(II) adsorption onto the hydrochar better [49]. Based on the Langmuir parameters, UA_char has the highest Pb(II) adsorption capacity (Table 3), suggesting the positive effect of CO_2_ activation on Pb(II) adsorption capacity.

**Table 3:** Langmuir and Freundlich isotherm parameters for Pb(II) adsorption on U_char, UA_char, EN_char, and EA_char at 25 °C.

Sample	Langmuir model			Freundlich model		
	*q*_m_ (mg g^−1^)	*K*_L_ (L mg^−1^)	*R*^2^	*n*	*K*_F_ (mg g^−1^)	*R*^2^
U_char	3.60	0.04	0.9770	1.57	0.25	0.9994
UA_char	12.88	3.42	0.9999	5.84	6.47	0.7991
EN_char	2.15	0.14	0.9302	2.21	0.42	0.8028
EA_char	12.36	11.19	0.9999	6.97	7.35	0.8752

### Enhancing adsorption performance via alkali treatment

The Pb(II) adsorption capacity of UA_char is greatly restricted by the high ash content, which is less compared to that of similar biomass materials [50]. Generally, adsorption capacity for heavy metal is expected to be improved with acid washing via removing ash of bio-based char [51]. Here, the silica functional groups observed by FT-IR suggests that the ash in the hydrochar contains substantial amount of silica, which is unlikely to be removed with regular acids. Therefore, UA_char is selected as the reference and treated with a strong base, sodium hydroxide (NaOH), to remove silica-rich ash from hydrochar. After alkali treatment, UAW_char is obtained and evaluated on the adsorption capability.

**Fig. 4:**
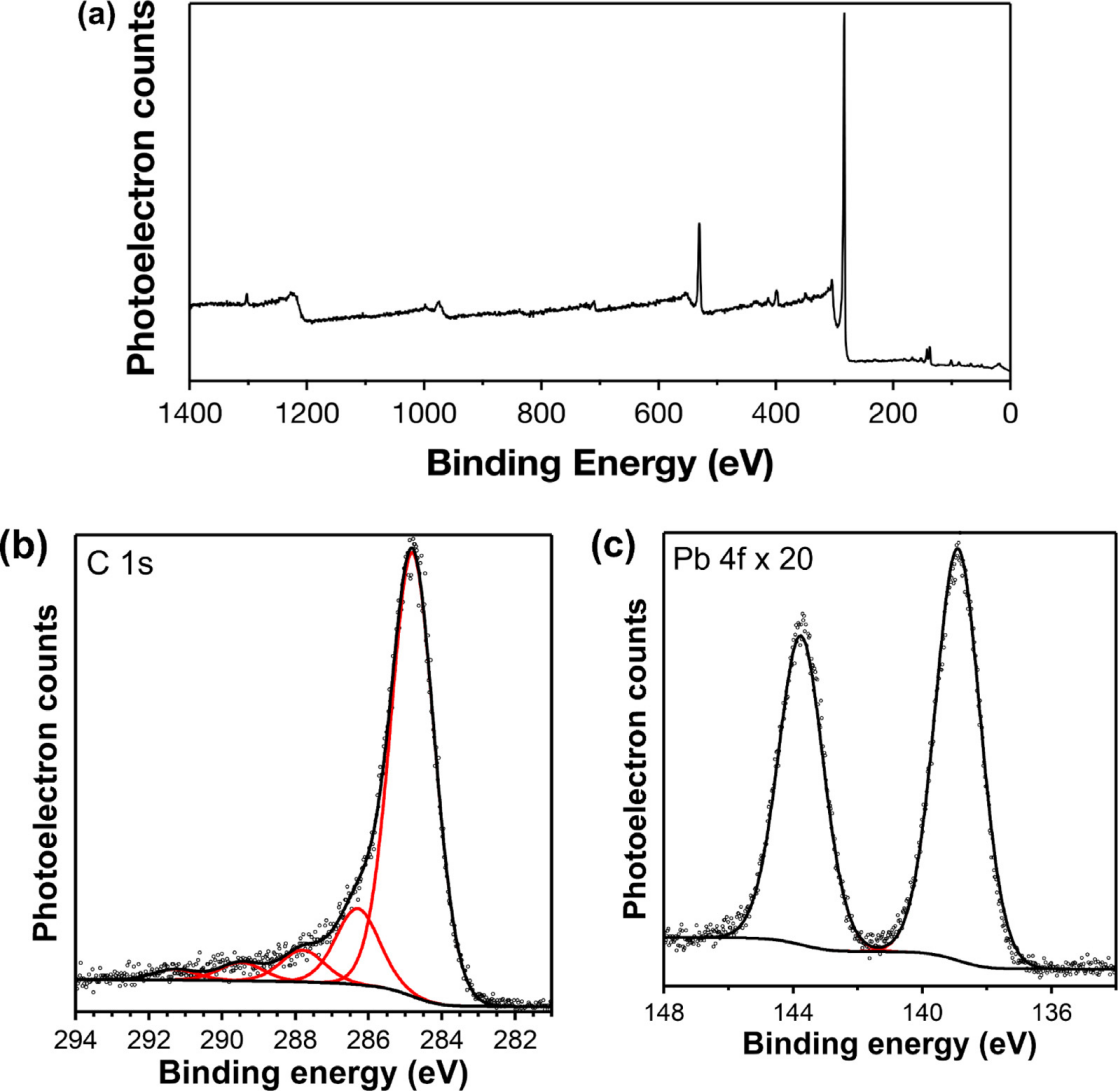
XPS spectra for UAWP_char. (a) Wide-energy XP survey spectrum; (b) C 1s spectrum; and (c) Pb 4f spectrum, which is magnified by a factor of 20 relative to the height of the C 1s region in (b).

To monitor Pb(II) adsorption on hydrochar surface, surface elemental composition of UAWP_char was characterized by XPS spectra and presented in Figs. 4 and S2. As shown in Fig. 4(a), the wide-energy survey scan is dominated by C 1s feature. Peak positions of all features in the high-resolution photoelectron regions are calibrated such that the primary feature in the C 1s region is centered at 284.8 eV. The C 1s region (Fig. 4(b)) contains four additional higher-binding energy features centered at 286.3 (C—O), 287.8 (*C* = *O*), 289.5 (*O* = *C*—O), and 291.3 (π-π*) eV [52], respectively. A doublet of peaks describing features in the Pb 4f region is displayed in Fig. 4(c), in which the Pb 4f_7/2_ and Pb 4f_5/2_ peaks are centered at 138.9 and 143.8 eV [53], respectively. The ratio (0.068) of Pb 4f to C 1s is quantified from experimentally determined peak areas corresponding to the XPS spectra. Furthermore, high-resolution Mg 1s, Na 1s, O 1s, and Ca 2p XPS spectra for UAWP_char are illustrated in Fig. S2, which are magnified in accordance with the scaling in Fig. 4. Appearance of Pb 4f feature proves the effective adsorb of Pb(Ⅱ) on the surface of algae-derived hydrochar.

**Fig. 5:**
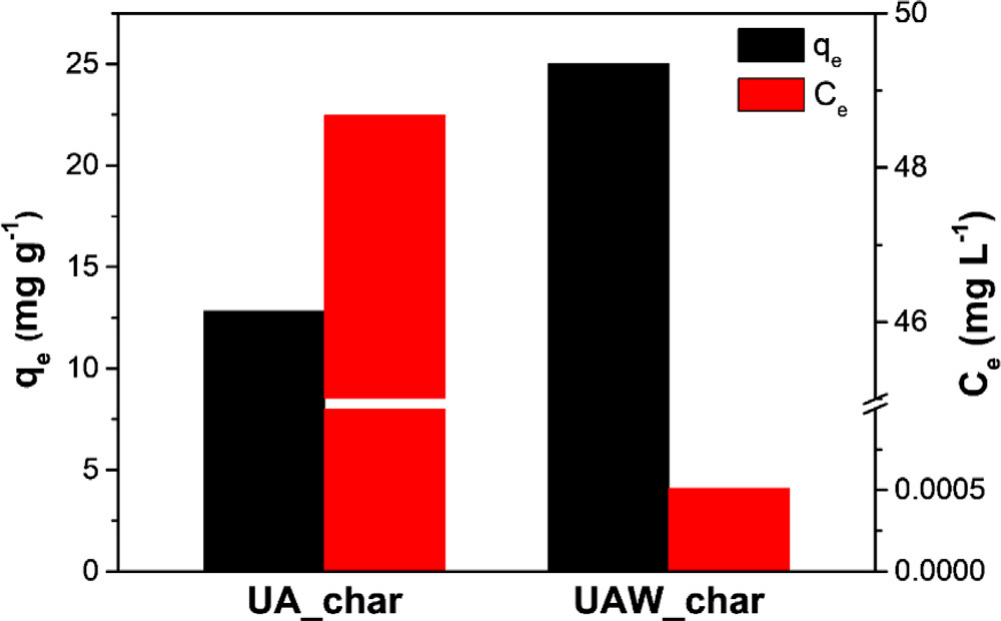
Pb adsorption capacities (*C*_e_: equilibrium Pb concentration, and *q*_e_: adsorption capacity at *C*_e_) for UA_char and UAW_char.

As rinsed with alkali solution, the Pb(II) adsorption capacity of UAW_char is improved from 12.83 mg g^−1^ for UA_char to 25.00 mg g^−1^ (Fig. 5), which is more comparable to those from other studies (Table 4). Therefore, the optimum adsorption condition is determined as CO_2_ activation, followed by alkali treatment. Interestingly, the equilibrium Pb(II) concentration, *C*_e_, reaches as low as 5.1×10^-4^ mg L^−1^, which is substantially less than from most previous studies (Table 4). This suggests an impressive potential of hydrochar studied here for meeting the Pb discharge requirements of World Health Organization [54].

**Table 4:** Equilibrium Pb concentration (*C*_e_) in the literature.

Biomass source	*q*_e_ (mg g^−1^)	*C*_0_ (mg L^−1^)	Pb(II) solution volume (L)	Added adsorbent mass (g)	Removal efficiency (%)	*C*_e_ (mg L^−1^)	Ref.
UAW_char	25.00	100	0.025	0.1	100	5.1×10^-4^	This work
Cauliflower leaves	72.76	200	0.2	0.5	–	18.1	[55]
Grape pomace	28	100	0.05	0.025	–	86	[13]
Prosopis africana shell (HTC)	8.5	10	0.05	0.05	–	1.5	[49]
Prosopis africana shell (pyrolysis)	7.5	10	0.05	0.05	–	2.5	[49]
Watermelon peel	–	1	0.05	0.01	45	0.55	[2]
Coconut shell	–	100	0.25	1	54.3	45.7	[56]

## Conclusion

In this work, hydrochar generated from wastewater-grown algae-to-biofuel HTL process is evaluated in terms of adsorption capability for Pb(Ⅱ). Compared with Soxhlet extraction, CO_2_ activation is a more effective method to remove volatile compounds. Notably, with alkali treatment used on the ash-enriched hydrochar, the adsorption capacity of Pb(Ⅱ) is enhanced by 100% compared with untreated hydrochar. Furthermore, the equilibrium concentration of Pb(Ⅱ) is measured at 5.1×10^-4^ mg L^−1^ after adsorption, which meets a stringent requirement on wastewater discharging by World Health Organization. It should be noted that other heavy metal ions are needed to be considered in the practical situation due to the existence of multiple ions in the wastewater. Our work is limited in the bench scale experiment; thus, the next step is to set up a scale-up experiment and take techno-economic analysis into consideration.

## Declaration of Competing Interest

The authors declare that they have no conflict of interest.

## Appendix Supplementary materials

Image, application 1
