# Supplementary material for: Waste-to-wealth application of wastewater treatment algae-derived hydrochar for Pb(II) adsorption
Source: MethodsX. 2021 Feb 7;8:101263. doi: 10.1016/j.mex.2021.101263 (PMC8374291; doi:10.1016/j.mex.2021.101263)
Supplement: Supplementary file 1 [file mmc1.docx]

**Supplementary material**

**Protocol Article**

**Waste-to-wealth application of wastewater treatment algae-derived hydrochar for Pb(II) adsorption**

Jiuling Yu,^a^ Tianbai Tang,^a^ Feng Cheng,^a^ Di Huang,^a^ Julia L. Martin,^b^ Catherine E. Brewer,^a^ Ronald L. Grimm,^b^ Meng Zhou,^a^ Hongmei Luo^a,*^

^a^ Department of Chemical and Materials Engineering, New Mexico State University, Las Cruces, New Mexico 88003, USA.

^b^ Department of Chemistry and Biochemistry; Life Science and Bioengineering Center; Worcester Polytechnic Institute, 100 Institute Road, Worcester, Massachusetts 01609, USA.

* Corresponding author. E-mail: [hluo@nmsu.edu](mailto:hluo@nmsu.edu)

## Characterization

- Boehm titration method

Oxygen-containing acidic groups on the surface of hydrochar was characterized by Boehm titration method. Briefly, 0.25 g of each hydrochar was mixed with 25 mL alkaline solutions, such as 0.01 M NaOH, 0.01 M NaHCO_3_ or 0.005 M Na_2_CO_3_. The mixtures were sealed well and kept under magnetic continuous stirring for over 12 h at room temperature. And then, the solutions were filtered and titrated with 0.005 M H_2_SO_4_ to quantify the amount of excess base. The assumption of Boehm titration method is that NaOH can neutralize carboxylic, lactonic and phenolic groups on the carbon surface, while Na_2_CO_3_ can neutralize both carboxylic and lactonic groups. But only carboxylic groups can be neutralized by NaHCO_3_ [1, 2].

- X-Ray photoelectron spectroscopy (XPS)

A PHI 5600 XPS system acquired all photoelectron spectra as described previously [3]. Acquisitions included wide-energy survey scans as well as high-resolution (23.5 eV pass energy, 50 meV step size, and a 50 ms-per-step dwell time) scans of the Pb 4f, C 1s, Ca 2p, O 1s, Na 1s, and Mg 1s regions for UAWP_char. Analysis of the char sample required neutralization with an ion flood gun. Post-acquisition fitting utilized an in-house-developed LabVIEW-based program. Spectral background shapes that describe the contribution from inelastically scattered electrons include a linear background, Shirley-shaped background [4], or the integration of a Tougaard shape based on B = 2900 eV^2^ and C = 1643 eV^2^ within a universal function that is scaled to the height of the photoelectron data [5]. A pseudo-Voight-style function, GL(x), describes each peak shape where x nonlinearly scales from 0 as a pure Gaussian form to 100 as a pure Lorentzian shape [6]. Optimization routines utilize the built-in LabVIEW implementation of the Levenberg–Marquardt algorithm for multiparameter fitting.

Fitting of the Pb 4f, C 1s, and O 1s utilized a Shirley-shaped background and GL(30) functional peak shapes. Fitting of the Mg 1s region utilized a linear background and GL(30) functional peak shapes. When fitting of the C 1s, O 1s, or Mg 1s regions necessitated multiple fit peaks, they were constrained to have identical full width at half max values.

Quantification of Pb 4f-to-C 1s ratio utilized sensitivity-factor-corrected peak areas which were defined as the quotient of the raw peak area and the respective sensitivity factor. We employed instrument-specific sensitivity factors for photoelectron collection at 90° with respect to the incident X-ray beam. Factors for Pb 4f and C 1s were 6.968 and 0.296, respectively. Quantification of this ratio utilized total peak area from all features in the relevant photoelectron regions.

**Table S1.** Inorganic element analysis of algal biomass [7].

| Element | Algae biomass (mg/kg) |
| --- | --- |
| Ca | 31770 - 35810 |
| K | 24780 - 27340 |
| Mg | 6371 - 6948 |
| S | 16440 - 18420 |
| P | 4630 - 5112 |
| Fe | 2414 - 2609 |
| Cu | 464 -548 |
| Mn | 233 - 256 |
| Zn | 183 - 203 |
| Na | 2379 |
| Al | 867 |
| Sr | 265 |
| B | 197 |
| As | 20 |
| Ba | 17 |
| Bi | 10 |
| Ni | 4 |
| V | 3 |


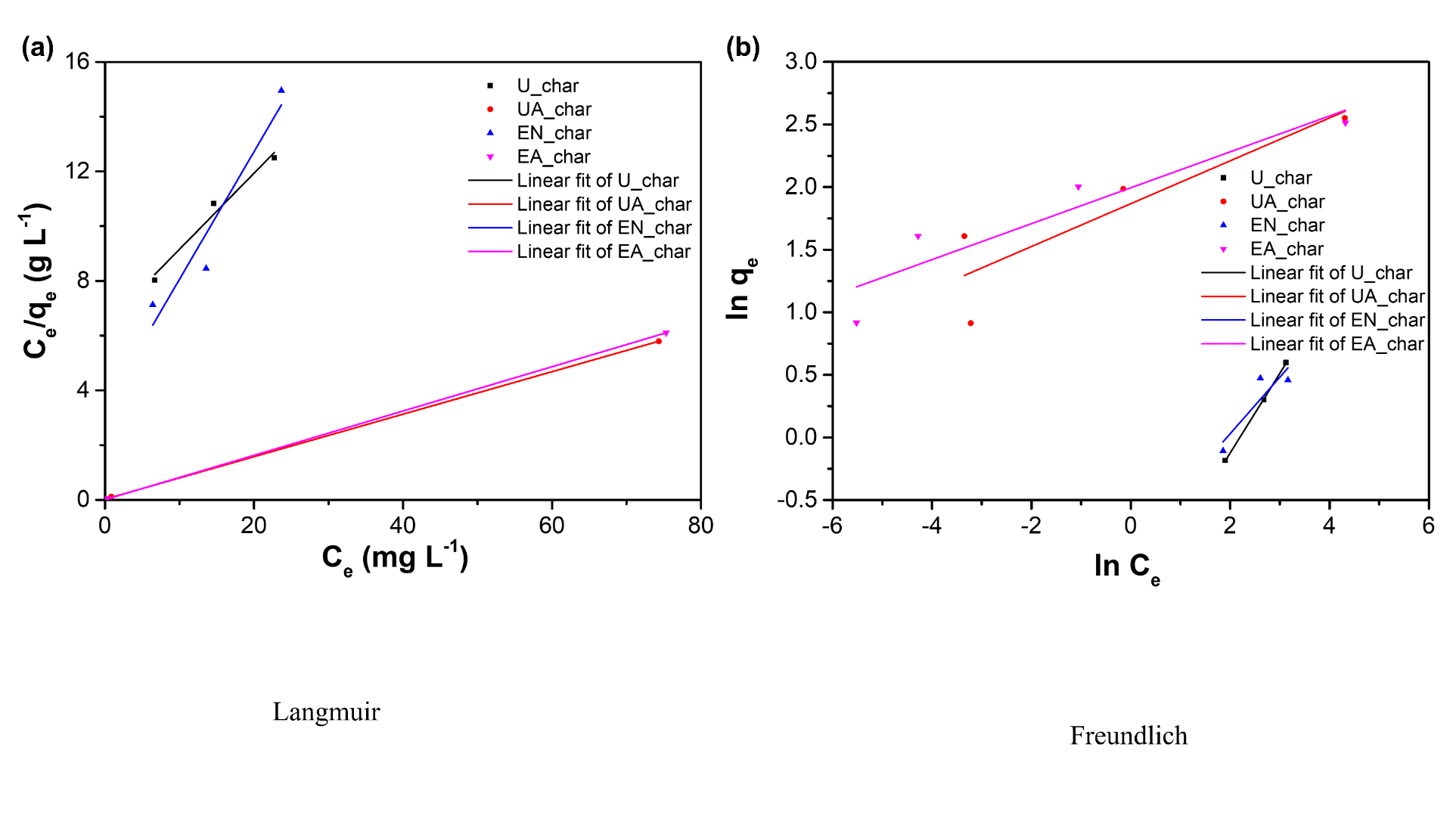

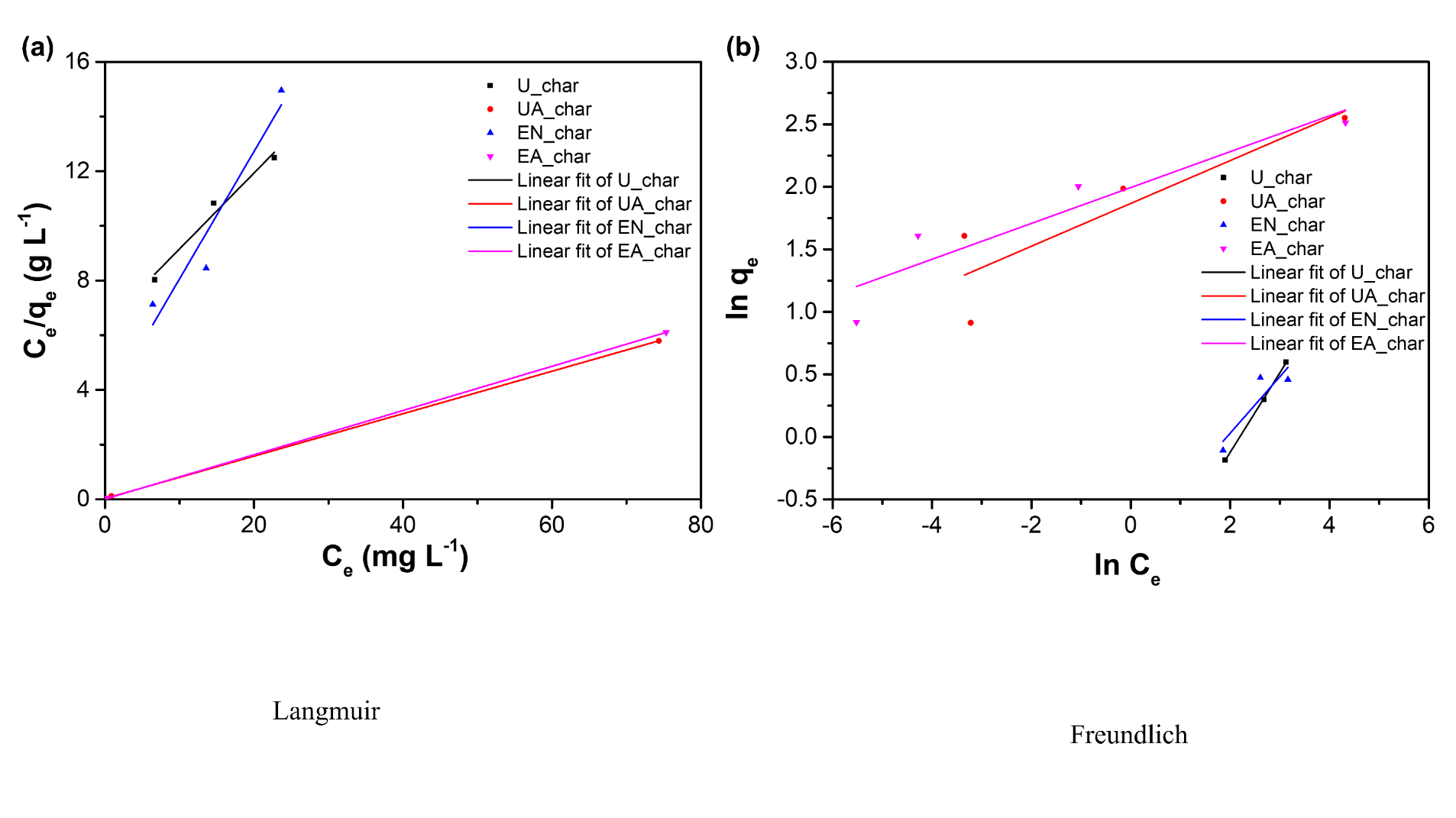


**Fig. S1.** (a) Linear Langmuir isotherm plots, and (b) linear Freundlich isotherm plots for Pb(Ⅱ) adsorption on U_char, UA_char, EN_char, and EA_char at 25 °C.


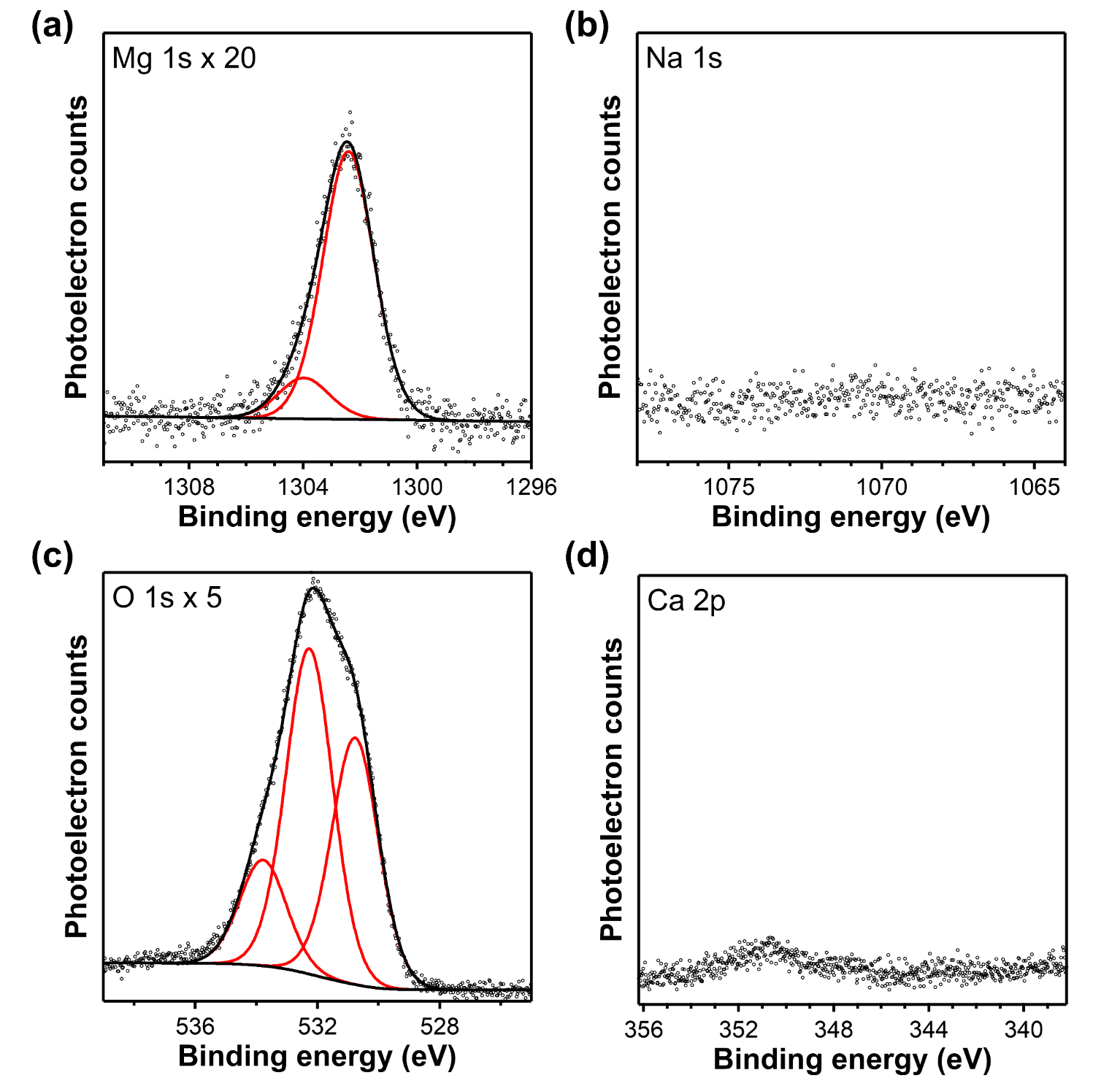

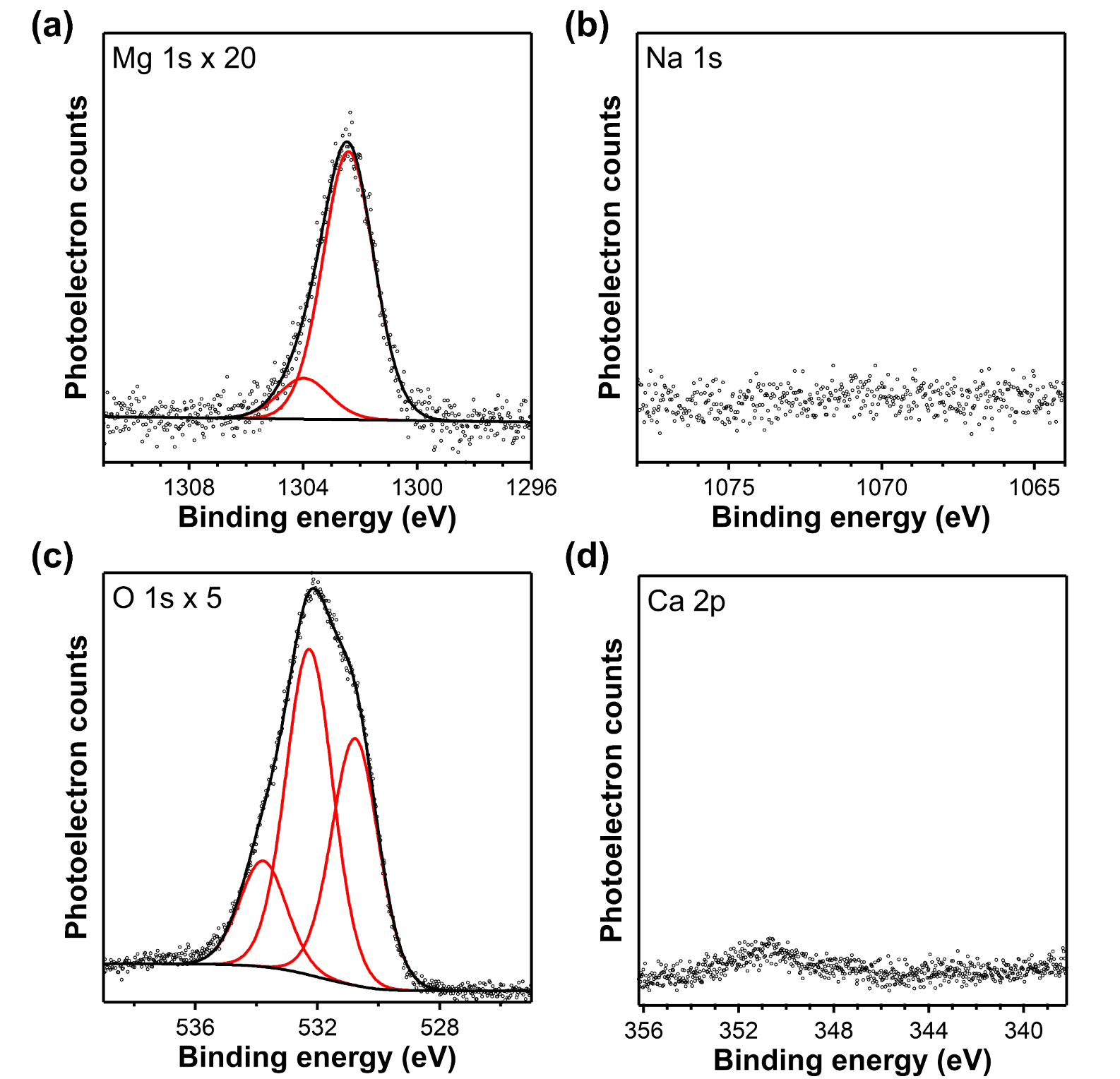

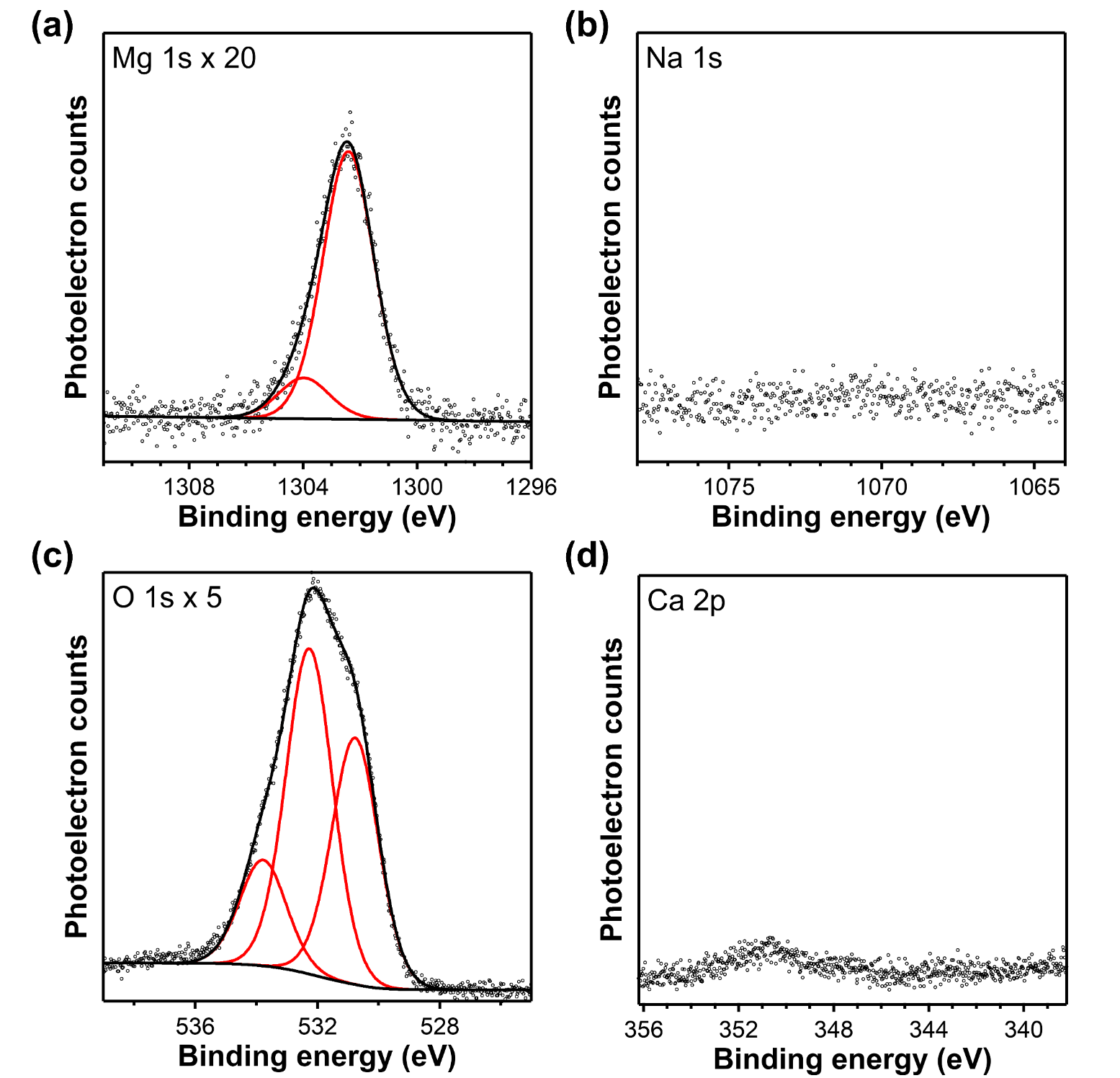

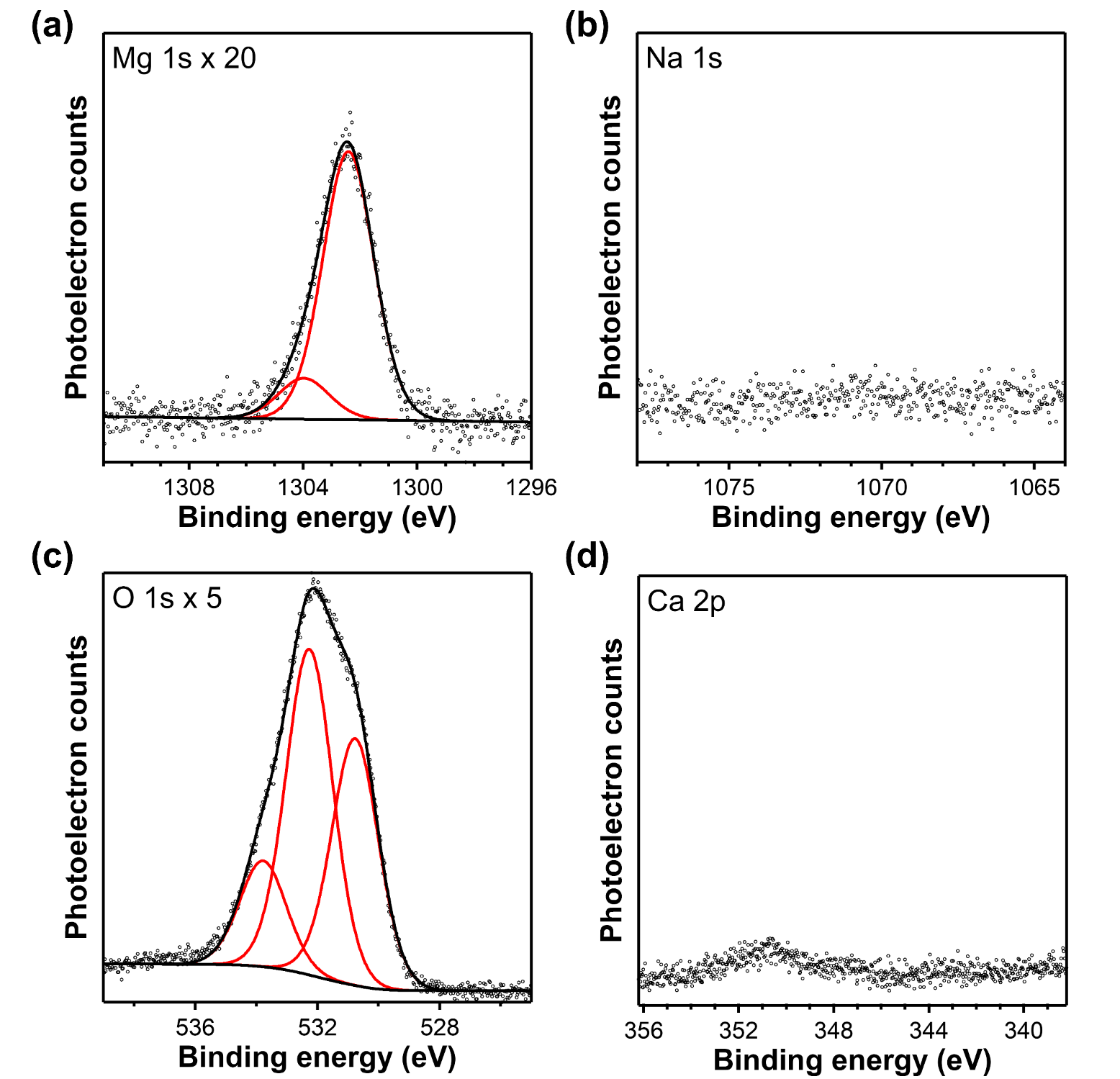


**Fig. S2**. XPS spectra for UAWP_char. (a) Mg 1s spectrum; (b) Na 1s spectrum; (c) O 1s spectrum; and (d) Ca 2p spectrum.

**References:**

[1] C. He, A. Giannis, J.-Y. Wang, Conversion of sewage sludge to clean solid fuel using hydrothermal carbonization: Hydrochar fuel characteristics and combustion behavior, Appl. Energy 111 (2013) 257-266.

[2] X. Zhu, Y. Liu, C. Zhou, G. Luo, S. Zhang, J. Chen, A novel porous carbon derived from hydrothermal carbon for efficient adsorption of tetracycline, Carbon 77 (2014) 627-636.

[3] A.D. Carl, R.E. Kalan, J.D. Obayemi, M.G.Z. Kana, W.O. Soboyejo, R.L. Grimm, Synthesis and characterization of alkylamine-functionalized Si(111) for perovskite adhesion with minimal interfacial oxidation or electronic defects, ACS Appl. Mater. Interfaces 9 (2017) 34377-34388.

[4] D.A. Shirley, High-resolution X-ray photoemission spectrum of the valence bands of gold, Phys. Rev. B 5 (1972) 4709-4714.

[5] C. Jansson, H.S. Hansen, F. Yubero, S. Tougaard, Accuracy of the Tougaard method for quantitative surface analysis. Comparison of the Universal and REELS inelastic cross sections, J. Electron Spectros. Relat. Phenomena 60 (1992) 301-319.

[6] J. Walton, P. Wincott, N. Fairley, A. Carrick, Peak fitting with CasaXPS: A Casa pocket book, Accolyte Science, 2010.

[7] H. Wang, Pretreatment of microalgae to reduce ash prior to hydrothermal liquefaction, New Mexico State University, 2018.
